# Mathematical Modeling of the Phoenix Rising Pathway

**DOI:** 10.1371/journal.pcbi.1003461

**Published:** 2014-02-06

**Authors:** Chad Liu, Chuan-Yuan Li, Fan Yuan

**Affiliations:** 1 Department of Biomedical Engineering, Duke University, Durham, North Carolina, United States of America; 2 Department of Dermatology, Duke University Medical Center, Durham, North Carolina, United States of America; Johns Hopkins University, United States of America

## Abstract

Apoptosis is a tightly controlled process in mammalian cells. It is important for embryogenesis, tissue homoeostasis, and cancer treatment. Apoptosis not only induces cell death, but also leads to the release of signals that promote rapid proliferation of surrounding cells through the Phoenix Rising (PR) pathway. To quantitatively understand the kinetics of interactions of different molecules in this pathway, we developed a mathematical model to simulate the effects of various changes in the PR pathway on the secretion of prostaglandin E2 (PGE2), a key factor for promoting cell proliferation. These changes include activation of caspase 3 (C3), caspase 7 (C7), and nuclear factor κB (NFκB). In addition, we simulated the effects of cyclooxygenase-2 (COX2) inhibition and C3 knockout on the level of secreted PGE2. The model predictions on PGE2 in MEF and 4T1 cells at 48 hours after 10-Gray radiation were quantitatively consistent with the experimental data in the literature. Compared to C7, the model predicted that C3 activation was more critical for PGE2 production. The model also predicted that PGE2 production could be significantly reduced when COX2 expression was blocked via either NFκB inactivation or treatment of cells with exogenous COX2 inhibitors, which led to a decrease in the rate of conversion from arachidonic acid to prostaglandin H2 in the PR pathway. In conclusion, the mathematical model developed in this study yielded new insights into the process of tissue regrowth stimulated by signals from apoptotic cells. In future studies, the model can be used for experimental data analysis and assisting development of novel strategies/drugs for improving cancer treatment or normal tissue regeneration.

## Author Summary

Apoptosis, or programmed cell death, is known to be important for embryogenesis, tissue homoeostasis, and cancer treatment. Furthermore, researchers have recently observed that apoptosis may promote wound healing and tissue regeneration, and accelerate undesired solid tumor regrowth after chemotherapy/radiation therapy. Mechanisms of apoptosis-induced tissue regrowth are related to a molecular network discovered recently in our lab. To quantitatively understand the kinetics of interactions of different molecules in this network, we developed a mathematical model and validated it by comparing the simulation results to experimental data reported in previous studies. To gain new insights into the process of tissue regrowth after inducing apoptosis, we used the model to simulate the effects of radiation on the production of a key growth stimulating factor, PGE2, in apoptotic cells. Additionally, we simulated how PGE2 production could be altered when cells were treated with different inhibitors. We expect that the new mathematical model can be used in future studies to facilitate design of better approaches to cancer treatment or normal tissue regeneration.

## Introduction

Apoptosis, or programmed cell death, is an important and tightly controlled process in mammalian cells [1]. However, not all cells in the same population undergo apoptosis when exposed to identical death signals [2], [3]. This “fractional killing” phenomenon is problematic in cancer treatment, but may be beneficial for wound healing since it has been observed that surviving cells in damaged tissues repopulate at a more rapid pace [4], [5], [6]. While there could be multiple factors that contribute to the rapid regrowth, one potential mechanism is that apoptotic cells may release signals that can promote proliferation of surrounding cells through the “Phoenix Rising” (PR) pathway discovered recently in our lab [5], [6]. This pathway may play important roles in both regeneration of damaged normal tissues and recurrence of tumors after chemotherapy/radiation therapy.

Wound healing in normal tissues is a complicated process that is time-dependent and requires coordination of different cells. While it is known that inflammation is the initial response to tissue damage, the exact cellular and molecular events in wound healing remain unclear. It has been generally assumed that factors released from damaged tissues mobilize and recruit stem and progenitor cells to the damaged site, where they proliferate, differentiate, and eventually replace the damaged tissue [6], [7]. Our previous studies have shown that two of the key molecular players in the initial response are caspase 3 (C3) and caspase 7 (C7), which are two proteases activated during the execution phase of apoptosis [5], [6]. Mice lacking either of these caspases are deficient in skin wound healing and liver regeneration [6]. The activation of C3 and C7 triggers a cascade of molecular events that lead to upregulation of prostaglandin E2 (PGE2), a growth-promoting signal that stimulates stem and progenitor cell proliferation and thus tissue regeneration.

Tumor recurrence often happens after chemotherapy and radiation therapy due to incomplete killing of tumor cells [8], [9]. Our previous studies have shown that apoptotic cells in the tumor mass can release signals to stimulate proliferation of remaining cells [5], [6]. Here, C3 in apoptotic cells is again a key regulator for the upregulation of signals that promote tumor regrowth.

**Figure 1:**
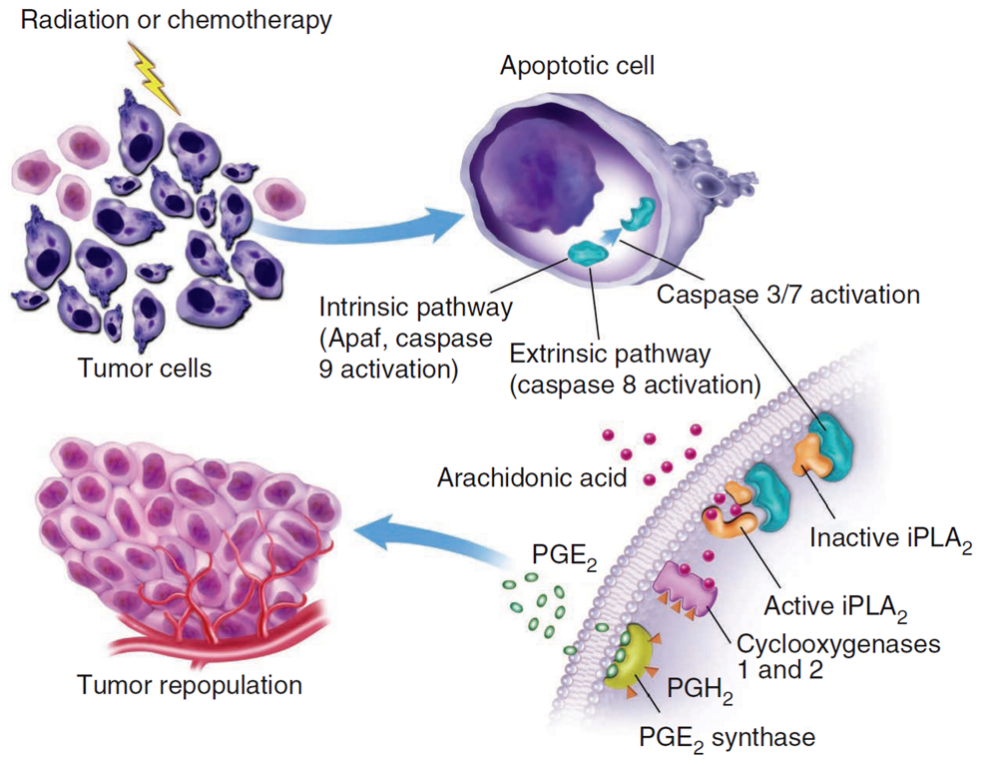
Schematic of the Phoenix Rising pathway. Radiation induces apoptosis of tumor cells, which causes release of PGE2 that can lead to rapid proliferation of remaining tumor cells. Reprinted from Ref. [5], with permission of Nature Publishing Group.

The PR pathway outlined in our previous studies involves a complicated network of molecular interactions [5], [6] (see also **Figure 1**). To understand the dynamics of these interactions, we developed a mathematical model that links the concentrations of activated C3, activated C7, and nuclear factor κB (NFκB) to the activity of PGE2 in the PR pathway. This type of “input-output” model, combined with experimental data, has been shown to be useful in understanding mechanisms of molecular events in cells [10]. Our model was built upon previous mathematical models for regulatory networks involved in apoptosis [1], [11] and arachidonic acid (AA) metabolism [12]. Our model was validated by comparing the predicted PGE2 concentrations in MEF and 4T1 cells at 48 hours after 10 Gray (Gy) radiation with the experimental data observed in previous studies [5], [6]. Using this model, we numerically simulated time-dependent changes in levels of key molecular players in the PR pathway after radiation therapy, and evaluated effects of C3 activation, C7 activation, C3 knockout, and cyclooxygenase-2 (COX2) inhibition on PGE2 production.

## Results/Discussion

**Figure 2:**
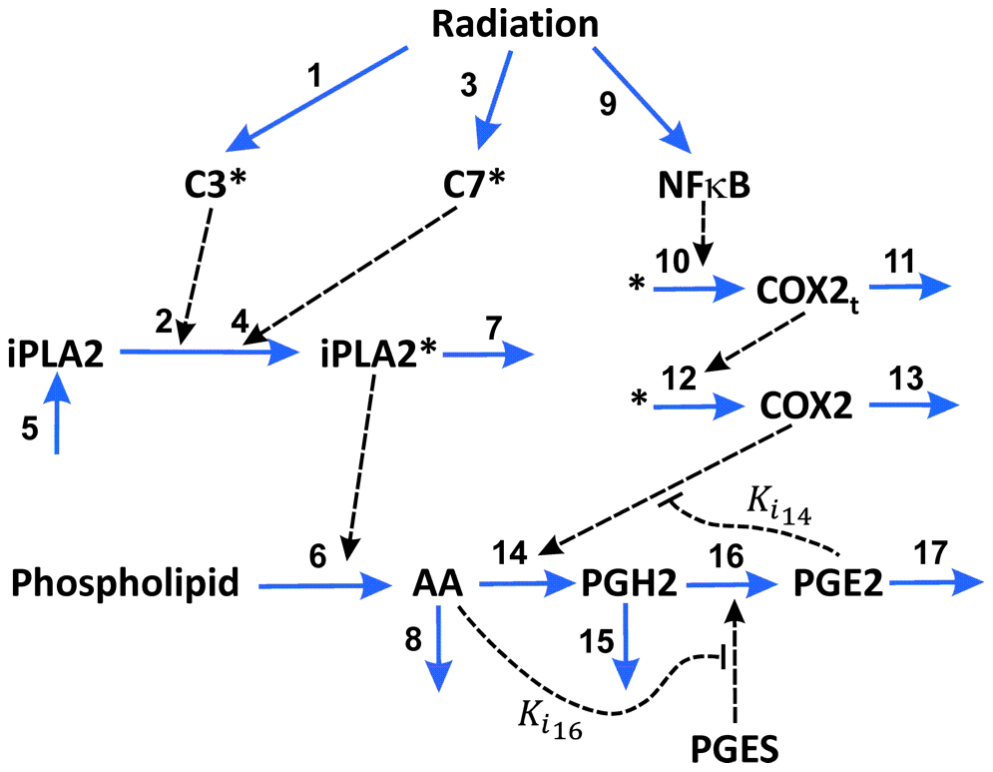
Mathematical model of the Phoenix Rising pathway. Seventeen molecular interactions in apoptotic cells were considered in the model. The input of the model was radiation-induced activation of C3, C7, and NFκB. Through the pathway, the activated molecules could result in secretion of PGE2 into the cell's microenvironment. The secreted PGE2 may promote proliferation of stem cells, progenitor cells, and tumor cells in surrounding regions. The mathematical model was used to simulate the dynamics of PGE2 synthesis and how inhibitions of different molecular players in the pathway affected PGE2 synthesis.

The PR pathway shown in **Figure 1** was modeled as a molecular network with seventeen key interactions. The model development was an iterative process, and involved various simplifications and assumptions (for details see the Materials and Methods section). The final outline of the model is shown in **Figure 2**. To validate the model, we compared numerically simulated [PGE2] with experimental data of [PGE2] reported in our previous study for wild-type MEF and 4T1 cells with/without radiation (see Figure 5b in Ref. [5]). It should be noted that two units are used for concentrations reported in this paper: pg mL^−1^ and µM. The former is used for [PGE2] in order to directly compare it with experimental data in the literature; the latter is used for concentrations of all other molecules in the model. The conversion factor for [PGE2] is 3.525×10^5^ pg mL^−1^ per µM. The maximal difference between model predictions and experimental data of [PGE2] was 9% (see **Table 1**), which was smaller than the uncertainties in the experimental data.

**Table 1:** Experimentally measured and numerically simulated [PGE2].^a^

	C3 KO MEF (0)	C3 KO MEF (10)	MEF (0)	MEF (10)	4T1 (0)	4T1 (10)
**Experimentally measured [PGE2] (pg mL^−1^)**	335^b^	525^b^	398	1730	285^b^	2100
**Numerically simulated [PGE2] (pg mL^−1^)**	333	525	388	1801	285	1910

a All experimental data were obtained from the literature, i.e., Figure 5b in Ref. [5]. Experimental data and numerically simulated [PGE2] were compared for three types of tumor cells: C3 knockout (KO) MEF cells, wild type MEF cells, and wild type 4T1 cells without (0) or with (10) 10-Gy radiation.

b The experimental data with the superscript were used to determine the values of [C3^*^]_0_, [C7^*^]_0_ and *k_3_*; and the data without the superscript were used to validate the model predictions.

**Figure 3:**
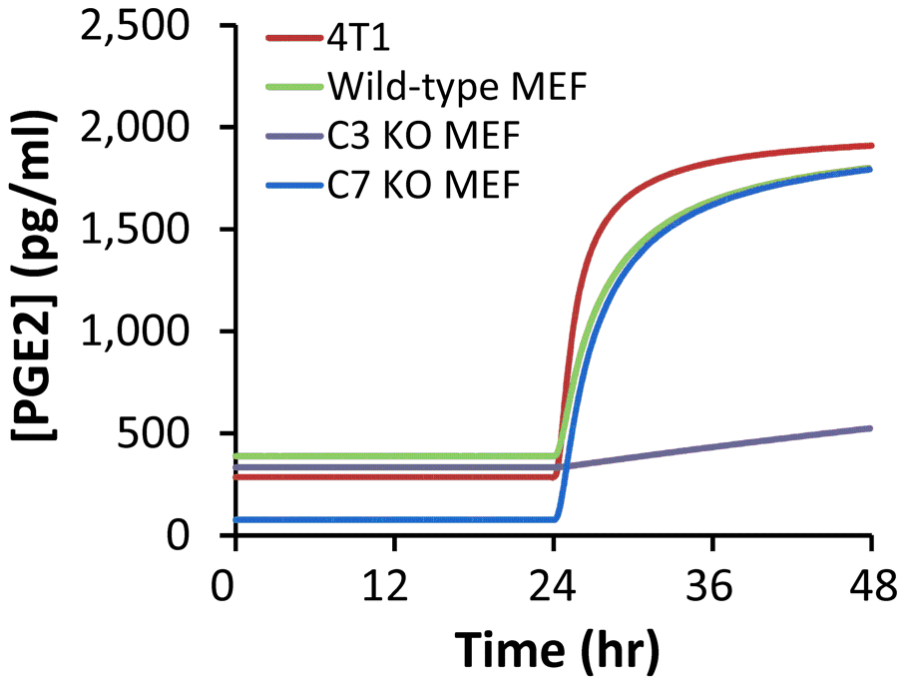
Model simulation of time dependent changes in [PGE2] after 10-Gy radiation of different tumor cells. The concentration profiles for wild type MEF and 4T1 cells were similar, but the production of PGE2 was significantly reduced in C3 knockout (KO) MEF cells. For C7 KO MEF cells, [PGE2] was low without radiation treatment. After 24 hours post radiation, [PGE2] started to increase, and quickly approached to the level in irradiated, wild type MEF cells.

**Figure 4:**
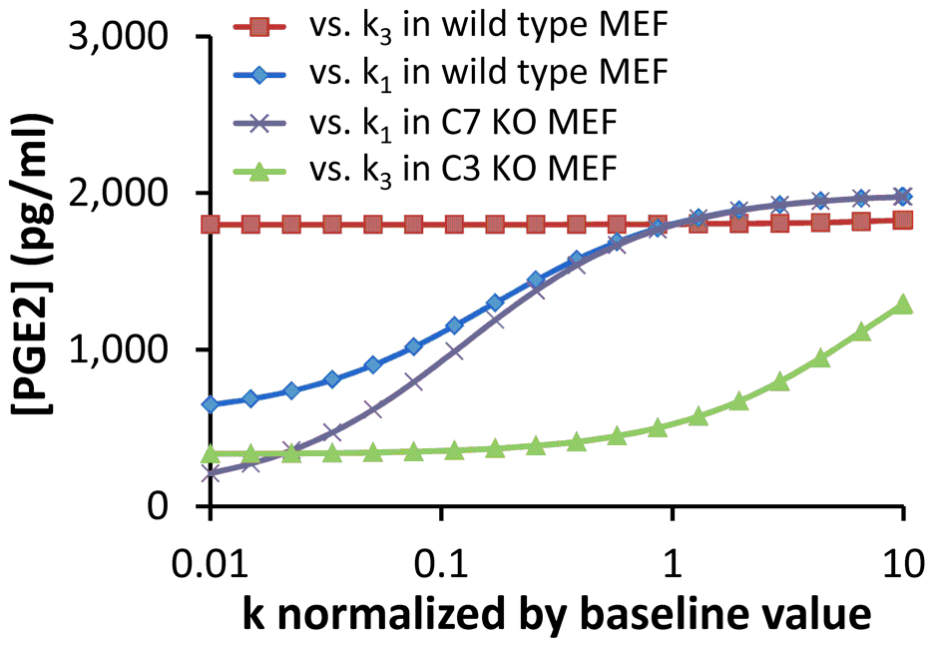
Dependence of simulated [PGE2] on k_1_ and k_3_ in MEF cells. The value of k_i_ (i = 1, 3) was normalized to its baseline shown in **Table 3**. [PGE2] was calculated at 48 hours after radiation. Four scenarios were simulated in the study: (i) dependence of [PGE2] on k_1_ when k_3_ was set at its baseline, (ii) dependence of [PGE2] on k_3_ when k_1_ was set at its baseline, (iii) dependence of [PGE2] on k_1_ in C7 KO cells, and (iv) dependence of [PGE2] on k_3_ in C3 knockout (KO) cells.

Using the model and the baseline values of the model constants listed in **Tables 2** **and** **3**, we examined the time-dependent changes in [PGE2] after 10-Gy radiation treatment. The numerical results shown in **Figure 3** demonstrated that there was a time delay of ∼24 hours in PGE2 production, due to slow activations of C3 and C7. After the delay, [PGE2] in wild-type MEF and 4T1 cells as well as C7 knockout (KO) MEF cells experienced a short, rapid increase phase, followed by a slow increase phase. In contrast, the simulated production of PGE2 in C3 KO MEF cells was slow and increased linearly with time between 24 and 48 hours. To investigate the dependence of [PGE2] on activation rates of C3 and C7, we simulated [PGE2] in MEF cells at 48 hours after radiation for a set of values of *k_1_* and *k_3_*. The numerical results shown in **Figure 4** demonstrated that [PGE2] was insensitive to changes in *k_3_* when *k_1_* was maintained at its baseline, but increased significantly with increasing *k_1_* when *k_3_* was maintained at its baseline, suggesting that PGE2 production was regulated mainly by the amount of activated C3 (C3^*^) but not activated C7 (C7^*^). To simulate effects of C3* or C7* deficiency on PGE2 production in irradiated cells, we assumed [C3*] (or [C7*]) be zero in C3 (or C7) knockout cells. The numerical results shown in **Figure 4** demonstrated that in C3 (or C7) knockout cells, activation of C7 (or C3) was important for increasing the production of PGE2. Taken together, our model predicted that activation of C3 was critical for PGE2 production whereas activation of C7 was important only in C3 knockout cells. This model prediction was consistent with experimental observations *in vivo* reported in our previous study [6], which showed that C3 deficiency could make MEF cells inefficient in promoting stem and progenitor cell proliferation. When both C3 and C7 were knocked out, our mathematical model predicted no PGE2 production in apoptotic cells. This prediction was also consistent with an experimental observation that lethally irradiated MEF cells with double knockout of C3 and C7 were less capable to promote stem or progenitor cell proliferation than those with only C3 knocked out [6].

**Table 2:** Simulation condition-independent rate and equilibrium constants.

	k	k-	K	K_i_
**2**	144 (min^−1^)^a^	-	11 (µM)^a^	-
**4**	26 (min^−1^)^a^	-	12 (µM)^a^	-
**5**	0.06 (min^−1^)^b^	0.09 (µM min^−1^)^a^	-	-
**7**	0.06 (min^−1^)^b^	-	-	-
**8**	0.06 (min^−1^)^b^	-	-	-
**10**	7.68×10^−4^ (µM min^−1^)^a^	-	5.2×10^−3^ (µM)^a^	-
**11**	0.6 (min^−1^)^b^	-	-	-
**12**	4.5 (min^−1^)^a^	-	-	-
**13**	0.06 (min^−1^)^b^	-	-	-
**14**	1000 (min^−1^)^c^	-	50 (µM)^c^	30 (µM)^c^
**15**	0.06 (min^−1^)^b^	-	-	-
**16**	3000 (min^−1^)^c^	-	160 (µM)^c^	0.3 (µM)^c^
**17**	0.06 (min^−1^)^b^	-	-	-

a Values for these rate and equilibrium constants were calculated based on the information in the literature. Details of the calculation are described in the Materials and Methods section.

b Values for these rate and equilibrium constants were assumed in this study.

c Values for these rate and equilibrium constants were obtained from the literature [12].

**Table 3:** Simulation condition-dependent model constants.^a^

	C3 KO MEF (0)	C3 KO MEF (10)	MEF (0)	MEF (10)	4T1 (0)	4T1 (10)
**[NFκB]_0_ (µM)**	0.1	0.1	0.1	0.1	0.1	0.1
[C3^*^]_0_ (nM)	0	0	0. 2	0. 2	0. 2	0. 2
[C7^*^]_0_ (nM)	6.0	6.0	6.0	6.0	3.8	3.8
***k_1_*** ** (µM min^−1^)**	0	0	0	2.8×10^−5^	0	6.8×10^−5^
***k_3_*** ** (µM min^−1^)**	0	3.24×10^−6^	0	3.24×10^−6^	0	3.24×10^−6^
***k_9_*** ** (µM min^−1^)**	0	3.47×10^−5^	0	3.47×10^−5^	0	3.47×10^−5^

a Details of the determination of these constants are described in the Materials and Methods section. The subscript “0” indicates steady state for unirradiated cells (0 Gy) or initial concentrations for irradiated cells (10 Gy). Values for these constants were determined for three types of tumor cells: C3 knockout (KO) MEF cells, wild type MEF cells, and wild type 4T1 cells without (0) or with (10) 10-Gy radiation.

**Figure 5:**
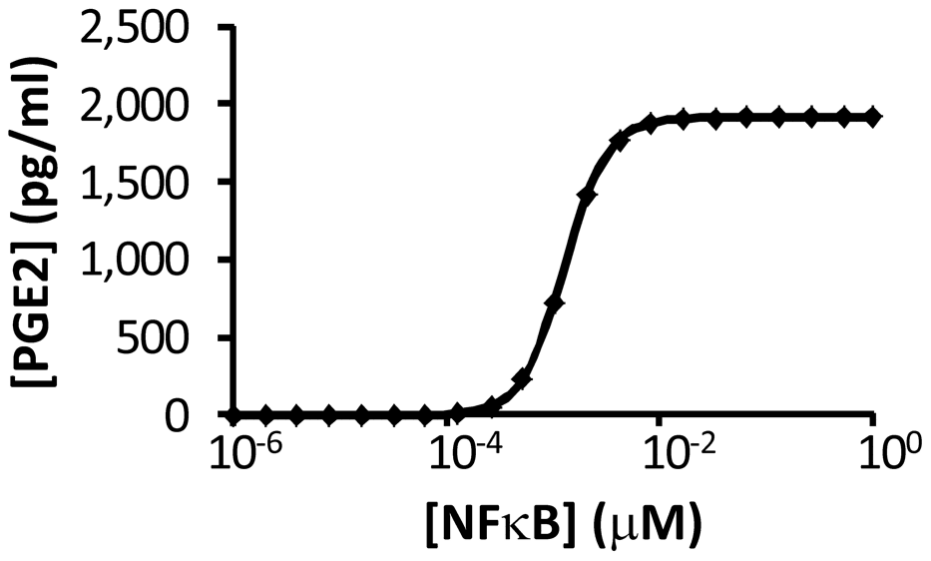
Dependence of simulated [PGE2] on [NFκB] in unirradiated 4T1 cells. For each level of [NFκB], the reported [PGE2] was determined at 48 hours when its level had reached a steady state.

Nuclear factor κB (NFκB) is critical for the regulation of PGE2 production through the control of COX2 expression (see **Figure 2**) [13]. To simulate effects of NFκB on PGE2 production, we fixed [NFκB] at different levels, and calculated the steady state [PGE2] in unirradiated 4T1 cells. The numerical results are shown in **Figure 5**; and similar results were also observed for unirradiated MEF cells (data not shown). These profiles demonstrated that the dependence of [PGE2] on [NFκB] variation was biphasic, which was controlled by the rate of COX2 transcription (see the equation for *v_10_*). When [NFκB] was <10^−4^ µM, the simulated [PGE2] was close to zero. When [NFκB] was increased from 10^−2^ to 1 µM, a two-order-of-magnitude change, the increase in simulated [PGE2] was minimal. This was because the increase in simulated concentration of COX2 mRNA (COX2_t_) was only 21%, which was too small to cause a significant increase in the simulated [PGE2]. In studies reported in the literature, [NFκB] in untreated cells is often higher than 0.1 µM [14], suggesting that the PR pathway was insensitive to radiation- or other tissue damage-induced increase in [NFκB].

**Figure 6:**
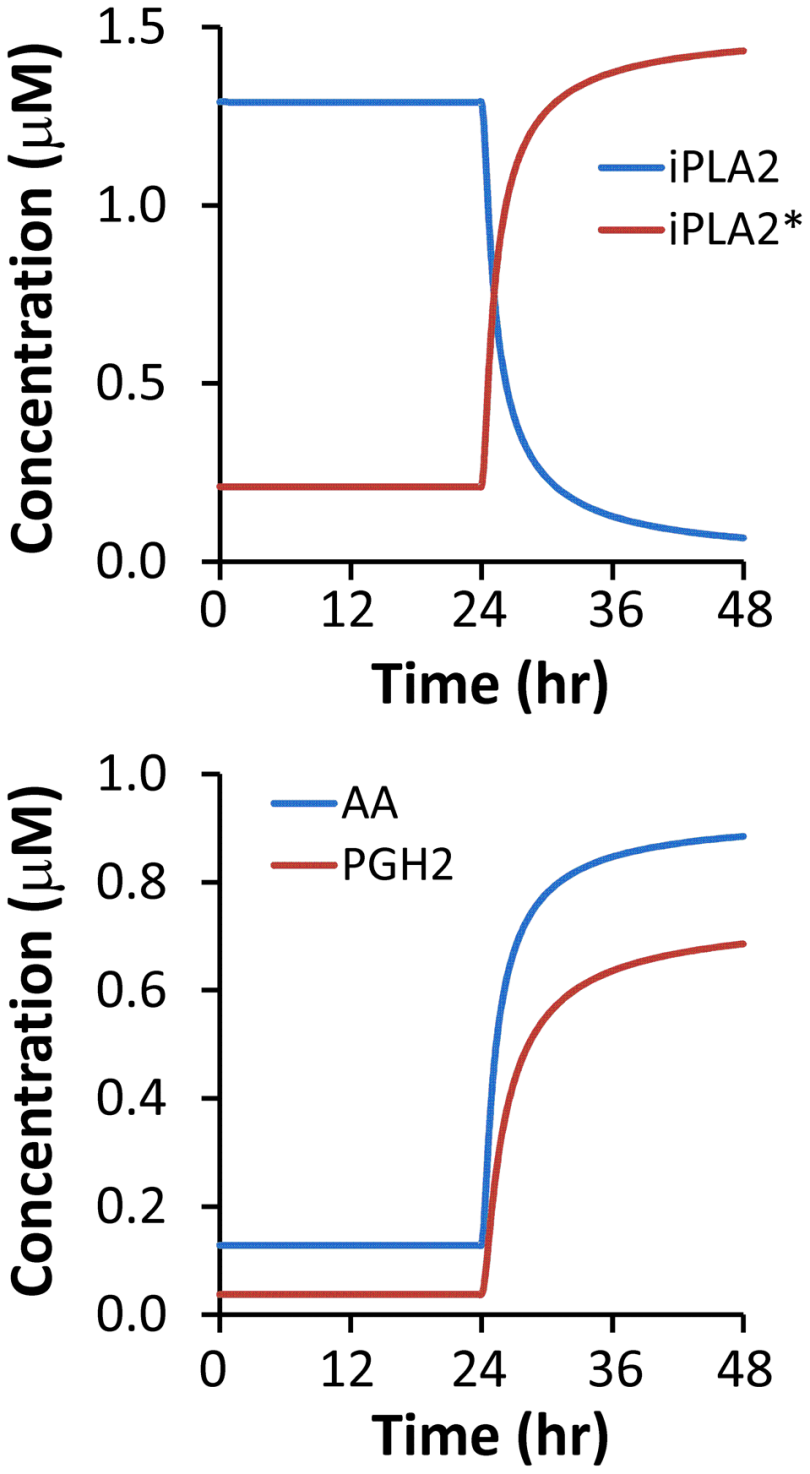
Model simulation of time-dependent changes in [iPLA2], [iPLA2^*^], [AA], and [PGH2] after 10-Gy radiation. The simulation was performed for wild type 4T1 cells.

Dynamic changes in numerically simulated concentrations of calcium independent phospholipase A2 (iPLA2), its active form (iPLA2^*^), arachidonic acid (AA), and prostaglandin H2 (PGH2) in 4T1 and MEF cells are shown in **Figures 6** **and** **7**. Differences in the corresponding profiles between MEF and 4T1 cells were small. The overall observation was that the simulated concentration of iPLA2^*^ in cells treated with 10-Gy radiation increased with time, which led to an increase in AA production from phospholipids. The numerically simulated [AA] profiles shown in **Figures 6** **and** **7** were quantitatively similar to those observed in previous experimental studies (see Figure 5a in both Ref. [5] and Ref. [6]).

**Figure 7:**
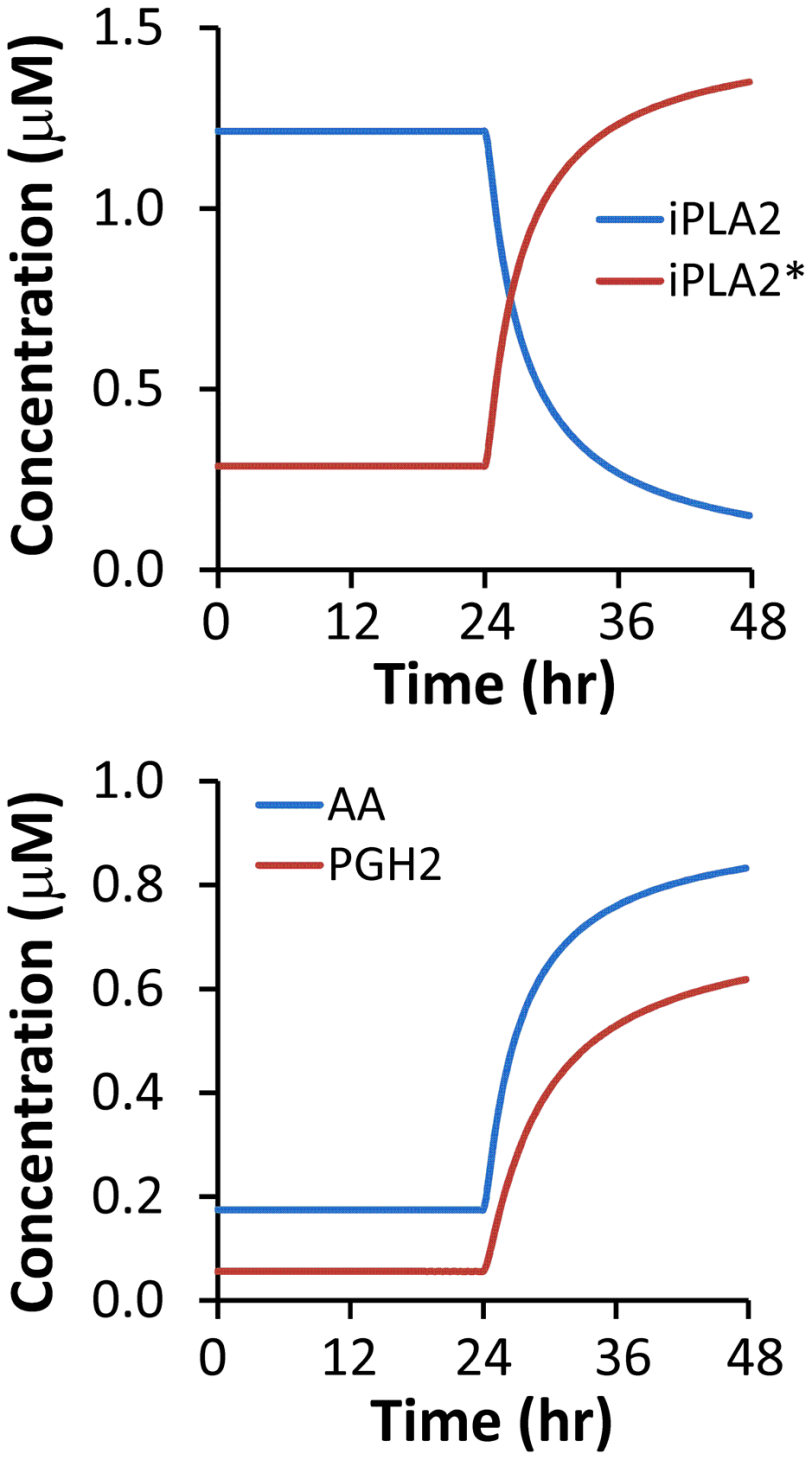
Model simulation of time-dependent changes in [iPLA2], [iPLA2^*^], [AA], and [PGH2] after 10-Gy radiation. The simulation was performed for wild type MEF cells.

**Figure 8:**
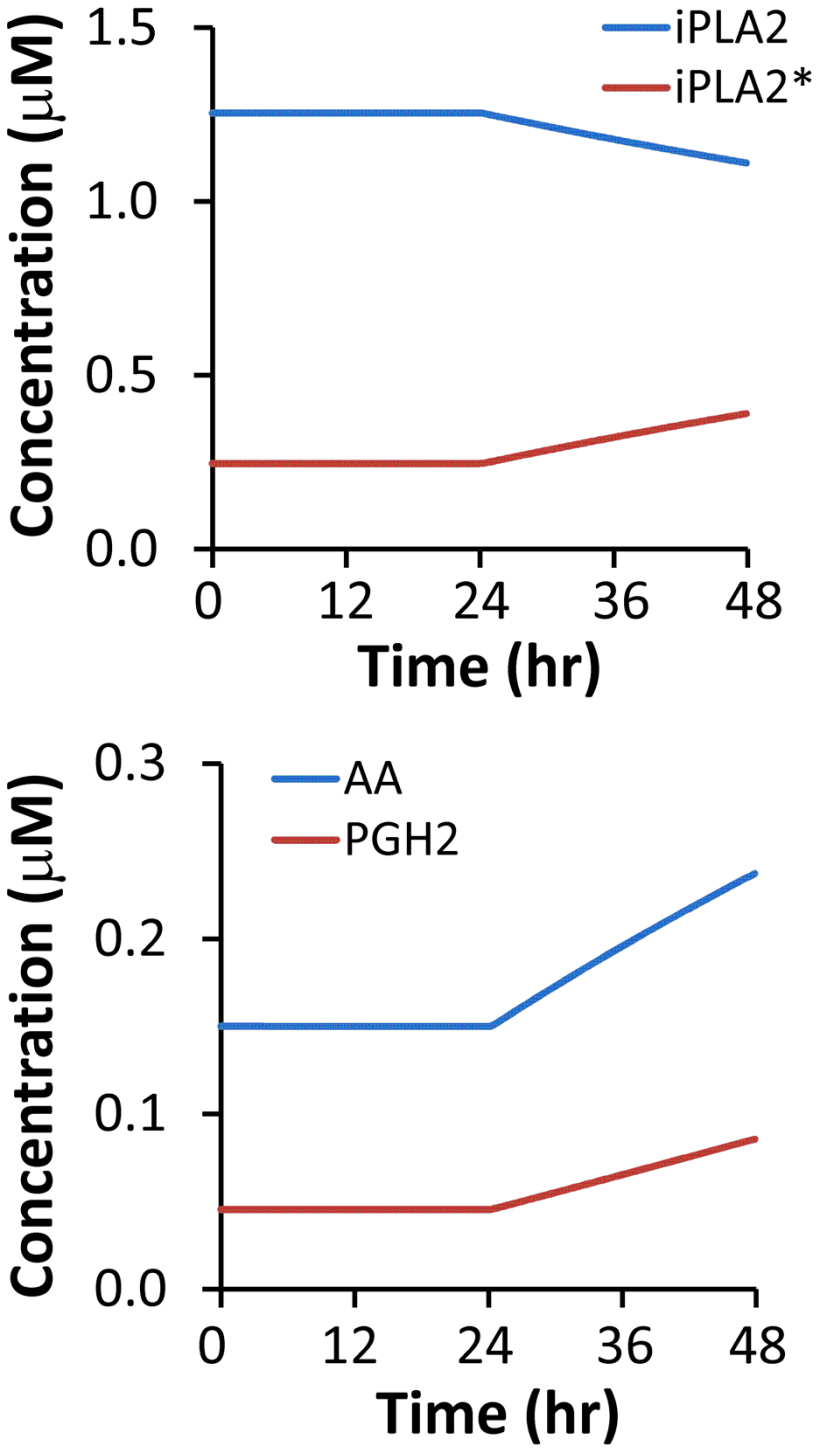
Model simulation of time-dependent changes in [iPLA2], [iPLA2^*^], [AA], and [PGH2] after 10-Gy radiation. The simulation was performed for C3 knockout MEF cells.

To simulate effects of C3 knockout on the PR pathway, we let [C3^*^] be zero. The simulated concentrations of iPLA2, iPLA2^*^, AA, and PGH2 in C3 knockout MEF cells are shown in **Figure 8**; and the dynamic change in simulated [PGE2] is shown in **Figure 3**. These results demonstrated that C3 gene knockout reduced the amount of activated iPLA2 by a factor of 3.5, thereby slowing the production of AA, which in turn reduced the levels of PGH2 and PGE2. Although the simulated concentrations of PGH2 and PGE2 would continue to increase with time beyond the 48-hour period, the increase may not happen in experimental studies. This is because our mathematical model did not consider cell proliferation nor the inactivation of C7* and NFκB that may happen in cells over time. More importantly, the cells may have already died/lysed after 48 hours.

**Figure 9:**
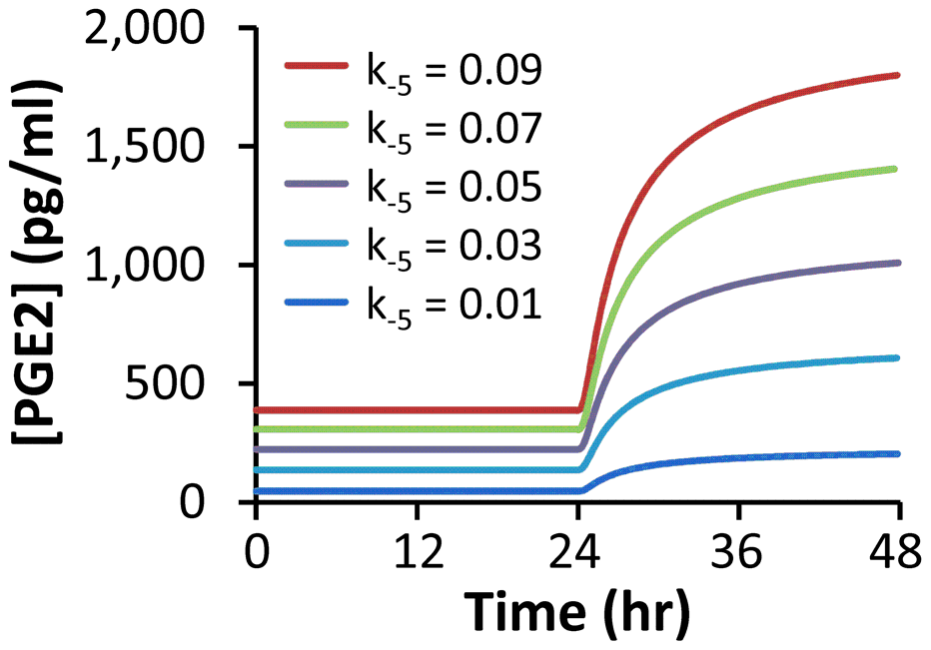
Effects of silencing iPLA2 expression on model simulated [PGE2] in wild type MEF cells at 48 hours post radiation. Experimentally, silencing gene expression can be achieved through treatment of cells with small interfering RNA molecules. In this study, silencing iPLA2 expression was mathematically modeled through reducing k_−5_ (unit: µM min^−1^), the rate of iPLA2 synthesis.

Effects of silencing iPLA2 expression on PGE2 production was simulated by reducing the rate of iPLA2 synthesis (k_−5_) in wild type MEF cells. The numerical results are shown in **Figure 9**, which demonstrated that the simulated [PGE2] at 48 hours post radiation decreased approximately linearly with decreasing *k_−5_*. This result was qualitatively consistent with our previous experimental observation where iPLA2 knockdown with shRNA reduced [PGE2] in MEF cells (Figure 5b in Ref. [6]).

**Figure 10:**
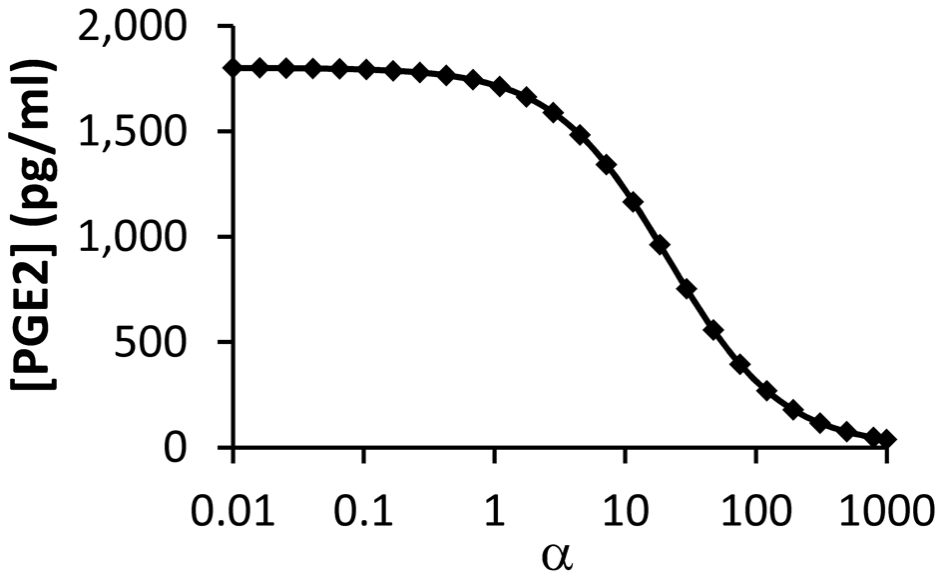
Effects of COX2 inhibition on model simulated [PGE2] in wild type MEF cells at 48 hours post radiation. Experimentally, COX2 inhibition can be achieved through treatment of cells with exogenous competitive inhibitors. In this study, the competitive inhibition was modeled through introducing a parameter, α, which was the ratio of inhibitor concentration versus equilibrium constant of binding between COX2 and its inhibitor. It can be observed that [PGE2] decreased rapidly with increasing the value of α.

Previous studies have shown that exogenous COX2 inhibitors can enhance efficacy of radiation therapy of cancer [15], [16], [17]. The mechanism of enhancement is likely to be related to the reduction in the release of growth-promoting signals, such as PGE2, from apoptotic cells. To simulate this mechanism, we introduced a parameter *α* to model competitive inhibition of COX2 (see the Materials and Methods section), and numerically simulated the dependence of [PGE2] on *α* at 48 hours post radiation. The simulation results shown in **Figure 10** indicated that [PGE2] decreased rapidly with increasing the value of *α*, suggesting that inhibition of COX2 could indeed reduce the production of growth-promoting signals in apoptotic cells.

We also examined the two negative feedback mechanisms (*Ki_14_* and *Ki_16_*) shown in **Figure 2** and their roles played in the regulation of PGE2 production *in silico*. We observed that removal of both negative feedback mechanisms (i.e., setting both *1/Ki_14_* and *1/Ki_16_* to zero) led to <2% increase in [PGE2], suggesting that these inhibitory mechanisms were weak and insignificant in the regulation of tissue regeneration after radiation treatment.

**Figure 11:**
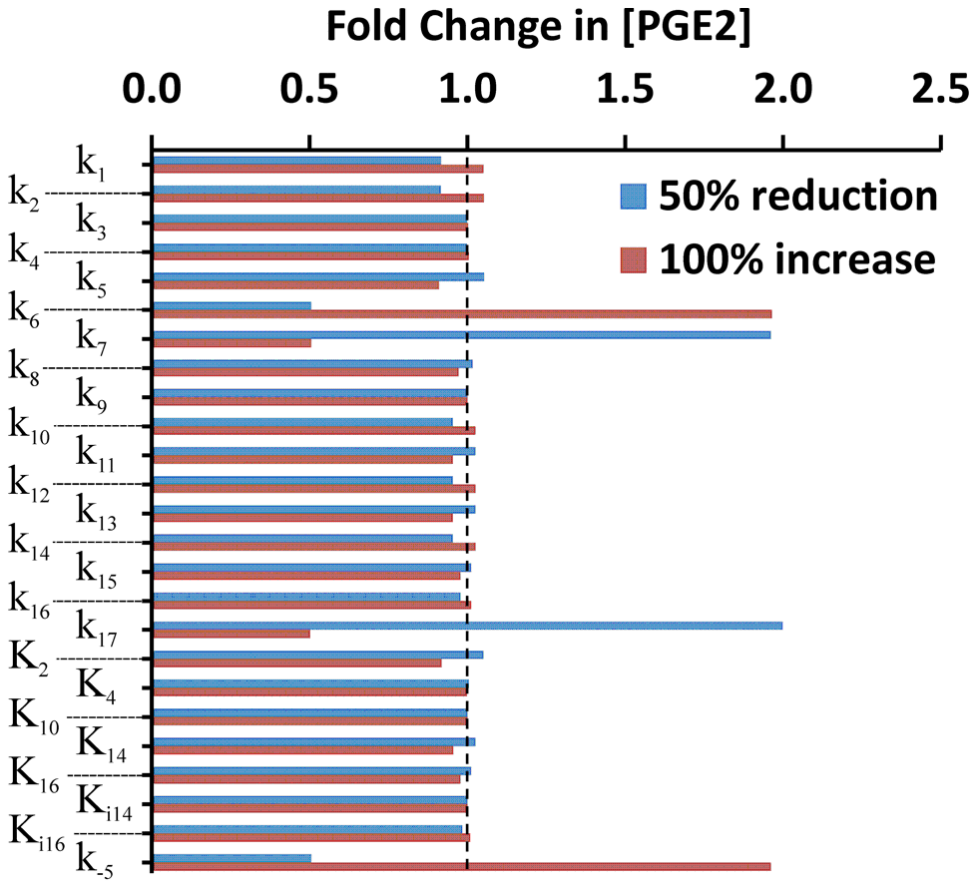
Sensitivity analysis of model simulated [PGE2] in wild type MEF cells at 48 hours post radiation. The value of each constant was either decreased or increased by a factor of two, relative to its baseline level shown in **Tables 2** and **3**. Effects of the variation in model constants on [PGE2], in terms of fold change in [PGE2], were calculated, relative to the baseline level of [PGE2]. It can be observed that [PGE2] was sensitive to variations in four rate constants: k_−5_, k_6_, k_7_, and k_17_. The dashed line indicates the level where the fold change was equal to unity, i.e., no change in [PGE2].

Finally, we performed a sensitivity analysis of model predictions. It was observed that [PGE2] was sensitive to only four rate constants: k_−5_, k_6_, k_7_, and k_17_, among all 25 rate and equilibrium constants (see **Figure 11**). Approximately, [PGE2] was proportional to changes in k_−5_ and k_6_, and reversely proportional to changes in k_7_ and k_17_. In the mathematical model, k_17_ determined the rate of PGE2 degradation, k_6_ and k_7_ determined the rates of AA production and degradation, respectively, and k_−5_ affected AA production indirectly through regulation of iPLA2 synthesis. Therefore, the analysis suggested that the simulated concentration of PGE2 was sensitive to its rate of degradation and the intracellular concentration of AA.

In summary, the mathematical model of the PR pathway yielded new insights into the process of growth-promoting signal production in apoptotic cells. While the model is limited by uncertainty in some parameter values and is only as good as the assumptions that were made, it provides useful information on the PR pathway. The model can be used to integrate experimental data obtained from different studies, adjust for cell-to-cell variability observed in different experiments, and determine sensitivity of the PR pathway to individual molecular interactions. The model can be further improved to serve as a tool for *in silico* experiments in future studies that may generate experimentally testable hypotheses, and facilitate development of novel strategies for improving cancer treatment and normal tissue regeneration.

## Materials and Methods

### A. Experimental determination of [C3^*^]

To determine [C3^*^], we analyzed digital images of Western blot gels reported in our previous study [5]. For each C3 or C3^*^ band in the images, we inversed its intensity and measured the total intensity over the entire area covered by the band, using the Gel Analysis function in ImageJ software. After background intensity subtraction, the total intensity data was used as a measure of the relative protein concentration. To determine the absolute concentration of C3 and C3^*^, we assumed that the concentration of C3 in unirradiated cells was 200 nM [11], and then used its band intensity to normalize those of C3^*^ under different experimental conditions. [C3^*^] was determined as the normalized image intensity of C3^*^ multiplied by 200 nM.

### B. Mathematical model

#### Model development

The PR pathway begins when a damaged cell activates its execution caspases (e.g., C3 and C7) to undergo apoptosis. The caspases then proceed to cleave/activate iPLA2, which binds to the membrane and catalyzes the hydrolysis of phospholipids to produce AA. AA is then converted to PGH2, catalyzed by COX2, which in turn is used to produce PGE2 catalyzed by prostaglandin E synthases (PGES) (see **Figure 1**) [5], [6], [18]. Finally, PGE2 is secreted into the environment of apoptotic cells to signal for cell replacements in damaged tissues.

To model the PR Pathway, we considered cell damage through treatment (e.g., radiation), which led to activation of C3, C7, and NFκB [5], [6], [19]. The activation processes have been modeled extensively in previous studies [1], [11], [20], [21], [22]. Thus, we used the activated forms of these molecules, i.e., C3^*^, C7^*^, and NFκB, as inputs for our model. The activated C3 and C7 would then trigger a cascade of reactions, which involved AA, COX2, iPLA2, PGE2, and PGH2 in the PR pathway shown in **Figure 1**. Meanwhile, the activated NFκB is critical for COX2 expression [13], and the latter is required for catalyzing PGH2 production from AA. The proposed network model also included two inhibitory reactions based on a mathematical model of AA metabolism, developed by Yang *et al*. [12], where PGES and COX2 could be inhibited by AA and PGE2, respectively. To simplify the model of the PR pathway, we did not consider interactions between lysyl oxidases (LOXs) and AA, which may lead to a reduction in the production of PGE2 [12]. Although the amount of reduction might be small, further studies are needed to elucidate roles of LOXs played in the PR pathway.

Based on all these considerations, we constructed a simple network model shown in **Figure 2**, which consisted of seventeen molecular interactions. In developing the mathematical model to recapitulate key experimental observations [5], [6], we made the following assumptions. *First*, chemical species shown in **Figure 2** behaved independently from other intracellular molecules that were not included in the model. *Second*, enzymatic reactions were governed by the Michaelis-Menten equation, except for the hydrolysis of phospholipids (see the description below), and they could be blocked by competitive inhibitors [12]. *Third*, PGES concentration was assumed to be time-independent within the simulation period (i.e., 48 hours) [12]. *Fourth*, NFκB is a transcription factor involved in the regulation of COX2 expression. The rate of transcription, i.e., COX2 mRNA (COX2_t_) synthesis, was assumed to be governed by the Hill equation [21]. The rate of translation from COX2_t_ to COX2 was assumed to be proportional to the concentration of COX2_t_. *Fifth*, iPLA2 is a housekeeping gene and constitutively expressed in cells under normal conditions [23]. Thus, its rate of synthesis was assumed to be constant. *Sixth*, the degradation of all chemical species considered in this model was assumed to be a first order reaction. *Finally*, the activated C3 and C7 were observed to be minimal but NFκB was clearly visible in Western blot analysis at 24 hours post radiation [5]. Therefore, the rate of NFκB production was assumed to be constant [24], and the rates of C3 and C7 activation were assumed to be proportional to the Heaviside step function of time, *H(t - 24)*, which is equal to 0 if *t<*24 hours and unity if *t≥*24 hours.

Phospholipid hydrolysis catalyzed by iPLA2^*^ has been modeled as a three-step reaction: (i) iPLA2^*^ adsorption to plasma membrane (M_B_), (ii) binding of adsorbed enzyme (iPLA2^*^-M_B_) to phospholipids (PS), and (iii) conversion of PS to reaction products, including AA [25], [26]. The final step in the reaction is very slow compared to the first one, indicating that the ratio of concentrations between iPLA2^*^ and iPLA2^*^-M_B_ is approximately equal to the equilibrium dissociation constant times the concentration of available adsorption sites on the membrane. Furthermore, it is known that the concentration of iPLA2^*^-M_B_ is approximately equal to that of iPLA2^*^-M_B_-PS [26], and the rate of AA production from PS is proportional to the concentration of iPLA2^*^-M_B_-PS. Assuming the amount of phospholipid hydrolyzed during the 48-hour period to be negligible compared to its total amount in a cell, we found that the rate of AA production was proportional to the total concentration of iPLA2^*^ in the cell (see the equation for *v_6_*). PGH2 production from AA is catalyzed by COX2, which can be competitively inhibited by either endogenous PGE2 or exogenous drugs [15], [16], [17]. To simulate effects of drug treatment on PGE2 production, a parameter α was included in the denominator of the equation for *v_14_*, which was proportional to intracellular concentration of the drug.

Based on the discussion above, the rates of reactions (*v_i_*, i = 1, 2, 3, …, and 17) shown in **Figure 2** were modeled as,
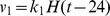


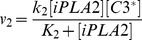


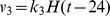


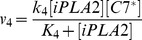


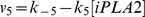


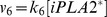


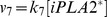


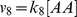


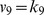


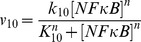


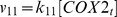


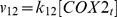


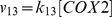


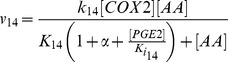


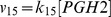


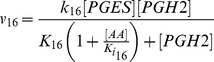


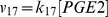



Mass conservation for the chemical species considered in the model required that
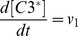


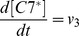


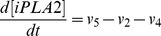


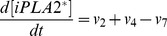


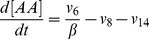


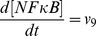


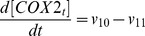


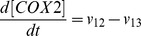


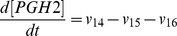


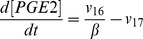



In these equations, all concentrations are defined as the number of moles per unit cell volume (V_c_) except for [AA] and [PGE2] because a fraction of these molecules produced in cells are secreted into extracellular medium, and it is the concentration in the medium that was measured in previous experiments. To directly compare model predictions with previous experimental data, [AA] and [PGE2] were therefore defined as the number of moles per unit volume of cells plus the medium (V_t_), which were close to the concentrations in the medium since the volume ratio (V_t_/V_c_), denoted by β, is significantly larger than unity, and there is no substantial difference in the concentrations between intracellular and extracellular spaces [27]. As a result, *v_6_* and *v_16_* in the mass balance equations for AA and PGE2 were corrected by a factor of β^−1^.

#### Initial conditions and numerical simulations

The initial conditions for all concentrations in irradiated cells were assumed to be equal to the steady state concentrations of the same chemical species in unirradiated cells. To determine the latter, the nonlinear differential equations described above were solved numerically, using the Dormand-Prince method (ODE45) in MATLAB, with all initial concentrations, except for [C3^*^], [C7^*^], and [NFκB], assumed to be zero. [C3^*^], [C7^*^], and [NFκB] in unirradiated cells were assumed to be time-independent, and the determination of their values, which were cell type-dependent, is discussed below.

### C. Determination of model constants

The baseline values of some model constants listed in **Tables 2** **and** **3** were assumed in this study. Specifically, the cell volume was assumed to be 1 pL, which means that 600 molecules per cell is 1 nM [28]. The degradation rates were assumed to be 0.6 min^−1^ for all mRNAs and 0.06 min^−1^ for all proteins [11], [21]. The baseline value of α is zero, i.e., there was no exogenous COX2 inhibitor unless indicated otherwise. *k_6_* was assumed to be 6×10^3^ min^−1^. [PGES] was assumed to be 0.5 µM [12]. The baseline values of other model constants were either obtained directly from the literature or estimated in this study based on model assumptions, experimental data reported in the literature, or values of similar constants used in previous mathematical models.

β. This constant depends on experimental conditions. For experiments reported in Ref [5], 2×10^5^ cells were cultured in 1 mL medium. Thus, the total volume of cells was 2×10^5^ pL; and β was approximately equal to 5000.

#### [C3^*^]_0_ and k_1_

[C3^*^] in unirradiated cells (i.e., [C3^*^]_0_) was too low to be detected accurately in the analysis of Western blot gel images described above. On the other hand, [C3^*^]_0_ was not necessarily equal to zero. Therefore, we adjusted [C3^*^]_0_ in simulations for unirradiated, wild type cells until the model predicted values of [PGE2] were consistent with the experimental data shown in **Table 1**. This procedure led us to choose [C3^*^]_0_ to be 0.2 nM or 0.1% of [C3], which was approximately 200 nM [11]. In C3 knockout cells, [C3^*^]_0_ was assumed to be zero. To determine the value of *k_1_*, we estimated concentrations of C3 and C3^*^ in irradiated cells through the analysis of Western blot gel images shown in the supplemental Figures 6a and 7a in Ref. [5] (see the procedure described above). Activation of C3 was observed only after 24 hours. Thus, *k_1_* was calculated as the difference between 0.2 nM and [C3^*^] data on Day 2, divided by 1440 min. The final results of *k_1_* for irradiated MEF and 4T1 cells are shown in **Table 3**. *k_1_* was assumed to be zero for C3 knockout cells and unirradiated cells.

#### [NFκB]_0_ and k_9_

[NFκB] in unirradiated cells (i.e., [NFκB]_0_) was assumed to be 0.1 µM in all types of cells in this study [14]. After 10-Gy radiation, the level of [NFκB] was assumed to increase linearly with time. The level of increase was observed to be one to four folds in 48 hours [24]. We chose the increase to be one fold for estimating the rate of [NFκB] increase since we observed in a preliminary simulation that the model output, i.e., [PGE2], varied insignificantly when the increase in [NFκB] was changed from one to four folds. As a result, *k_9_* for all irradiated cells was calculated to be 3.47×10^−5^ µM min^−1^ (see **Table 3**).

#### k_5_, k_−5_, and steady state concentration of iPLA2

Hydrolysis of phospholipids can be catalyzed by a superfamily of enzymes, called phospholipase A_2_ (PLA2) [29]. Among them, Ca^2+^ independent PLA2 (i.e., iPLA2) is an important housekeeping gene that is highly expressed in cells under normal conditions [23]. The level of its expression is on the same order of magnitude as that of the total PLA2 in cells, which has been assumed to be 1.5 µM at steady state [12]. Therefore, we assumed the steady state concentration of iPLA2, [iPLA2]_ss_, to be 1.5 µM in unirradiated cells. Although iPLA2 can be activated through cleavage to become iPLA2^*^, the rate of cleavage in unirradiated cells is likely to be negligible, compared to its degradation with the rate constant *k_5_*. Thus, we assumed that in these cells at steady state, *k_−5_ – k_5_*[iPLA2]_ss_≈0. Since the rate constant for degradation of all proteins had been assumed to be 0.06 min^−1^ in this study, *k_−5_* = 0.09 µM min^−1^.

#### k_2_, K_2_, k_4_, and K_4_

iPLA2 can be activated when it is cleaved by caspase 3 at Asp^513^ or Asp^733^ [30]. We assumed that iPLA2 activation by caspase 7 was also caused by its cleavage at an Asp site. For these enzymatic reactions, k_cat_/K_M_ has been determined to be 200,000 M^−1^ s^−1^ for caspase 3 and 33,000 M^−1^ s^−1^ for caspase 7 [31]. Talanian *et al*. have also measured k_cat_ and K_M_ for both caspase 3 and caspase 7 catalyzed reactions with various substrate sequences [32]. We chose the values of k_cat_ and K_M_, measured by Talanian *et al.*, based on the criterion that the k_cat_/K_M_ ratio must be consistent with the data reported in Ref. [31]. This requirement led to the choice of substrate sequence Asp-Glu-Val-Asp reported in Ref. [32], for which k_2_ and K_2_ were 144 min^−1^ and 11 µM, respectively, and k_4_ and K_4_ were 26 min^−1^ and 12 µM, respectively.

#### k_10_, K_10_, n, and k_12_

Following the assumptions made by Terry et al. [21], we assumed that expressions of different genes activated by NFκB had the same transcription and translation rates. Thus, the baseline values of k_10_, K_10_, and k_12_ were assumed to be equal to the maximal rate of NFκB induced transcription, NFκB half-maximal concentration, and rate of translation, respectively, reported in Ref. [21] (see **Table 2**). The Hill coefficient n in *v_10_* was assumed to be 2 [21].

#### [C7^*^]_0_ in unirradiated cells

[C7^*^]_0_ is the steady state concentration of C7* in unirradiated cells. There are no experimental data of [C7^*^]_0_. Thus, it was determined by fitting simulated [PGE2] to experimental data reported in our previous study for unirradiated C3 knockout (KO) MEF and wild-type 4T1 cells (see Figure 5b in Ref [5]). In the procedure, values of all other constants were fixed at the baseline levels shown in **Tables 2** **and** **3**. For unirradiated cells, *k_3_* = 0. Thus, [C7^*^] was time-independent, which was equal to [C7^*^]_0_. In predicting [PGE2] in unirradiated cells, *k_1_* = *k_9_* = 0, [C3^*^]_0_ was 0.2 nM for wild-type cells, and zero for C3 knockout cells, [NFκB] was 0.1 µM for both types of cells (see **Table 3**), and all other concentrations at t = 0 were zero. For C3 knockout MEF cells, [PGE2] at 48 hours was experimentally observed to be 335 pg mL^−1^ in our previous study [5] (see **Table 1**); and the best fit of the model prediction to this data required [C7^*^]_0_ to be 6.0 nM. For wild-type MEF cells, [C7^*^]_0_ was assumed to be 6.0 nM as well. For 4T1 cells, [PGE2] at 48 hours measured experimentally in our previous study [5] was 285 pg mL^−1^ (see **Table 1**), which was lower than that in MEF cells. Thus, [C7^*^]_0_ was reduced to 3.8 nM for the model prediction to fit the experimental data.

#### *k_3_* in irradiated cells

To determine *k_3_*, we first calculated the steady state concentrations in unirradiated cells, and then used them as the initial conditions for the differential equations described above. These equations were solved numerically with different values of *k_3_* to obtain [PGE2] at 48 hours post radiation. For C3 knockout MEF cells treated with 10-Gy radiation, the experimental data of [PGE2] was observed to be 525 pg mL^−1^ in our previous study [5] (see **Table 1**), and the best fit of the model prediction to this data required *k_3_* to be 3.24×10^−6^ µM min^−1^. For wild type MEF and 4T1 cells, *k_3_* could not be accurately determined through fitting the model prediction to the experimental data of [PGE2] since an increase in *k_3_* by three orders of magnitude led to only minimal increase (<5%) in [PGE2]. Thus, *k_3_* for all irradiated cells were assumed to be 3.24×10^−6^ µM min^−1^.
